# Smart Crack Control in Concrete through Use of Phase Change Materials (PCMs): A Review

**DOI:** 10.3390/ma11050654

**Published:** 2018-04-24

**Authors:** Branko Šavija

**Affiliations:** Microlab, Delft University of Technology, 2628 CN Delft, The Netherlands; b.savija@tudelft.nl; Tel.: +31-681-015-812

**Keywords:** concrete durability, smart concrete, phase change materials, microencapsulation

## Abstract

Cracks in concrete structures present a threat to their durability. Therefore, numerous research studies have been devoted to reducing concrete cracking. In recent years, a new approach has been proposed for controlling temperature related cracking—utilization of phase change materials (PCMs) in concrete. Through their ability to capture heat, PCMs can offset temperature changes and reduce gradients in concrete structures. Nevertheless, they can also influence concrete properties. This paper presents a comprehensive overview of the literature devoted to using PCMs to control temperature related cracking in concrete. First, types of PCMs and ways of incorporation in concrete are discussed. Then, possible uses of PCMs in concrete technology are discussed. Further, the influences of PCMs on concrete properties (fresh, hardened, durability) are discussed in detail. This is followed by a discussion of modelling techniques for PCM-concrete composites and their performance. Finally, a summary and the possible research directions for future work are given. This overview aims to assure the researchers and asset owners of the potential of this maturing technology and bring it one step closer to practical application.

## 1. Introduction

Reinforced concrete is a building material of choice for structures in challenging environments. Concrete is a highly durable building material and can have a long service life with little or no maintenance. When steel is embedded in concrete in order to take over tensile stresses, it is typically protected from corrosion by a passive film formed on its surface due to the high alkalinity of the concrete pore solution [1]. Under certain conditions, however, this passive film can break down, leading to reinforcement corrosion. Reinforcement corrosion can be caused by concrete carbonation [2] which leads to a drop in the pH value (below 9) and breaks down the passivation layer; or chloride ingress [3] which can locally break down the passive layer leading to pitting corrosion. Since steel corrosion is an expansive reaction, it will then lead to cracking and spalling of the concrete cover [4,5]. As a consequence, service life design guidelines require that the concrete cover to the reinforcement is of a certain dimension and quality [6,7]. These guidelines are, however, derived assuming that the concrete cover is uncracked. Reinforced concrete structures are, however, always cracked [8], due to a variety of reasons: mechanical loads, (restrained) shrinkage [9], thermal deformations [10], and freezing and thawing [11], among others. Most of these cracks do not pose a threat for the structural integrity of a structure. However, they may create durability issues: both chloride ingress [12] and carbonation [13] have been shown to advance much faster in cracked concrete, thereby leading to rapid steel depassivation and corrosion [14]. Consequently, there are numerous approaches proposed in the literature to minimize cracking in reinforced concrete structures.

**Figure 1:**
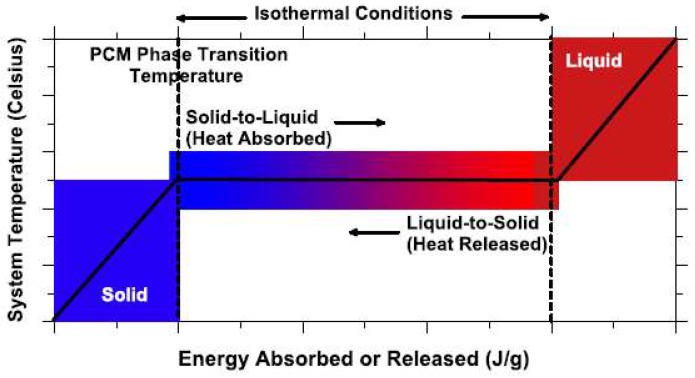
A schematic which illustrates the temperature–energy (heat) response of a phase change material (PCM) and the process therein [22].

Concrete is a quasi-brittle material that cracks at low strain levels [8]. One possibility for minimizing cracking is through the use of fiber reinforcement such as steel [15], polyvinyl alcohol (PVA) [16], or natural fibers [17]. If properly designed, these fibers are able to bridge cracks in concrete and reduce their width. A recent and more reactive approach is the use of self-healing concrete: once a crack forms, it is autonomously repaired by the material itself [18,19,20]. In this way, the durability of the structure is restored. In recent years, a new approach for reducing concrete cracking caused by thermal effects has been proposed: through incorporation of phase change materials (PCMs) in the concrete mix, temperature variations can be minimized thereby avoiding occurrence of thermal cracking. PCMs are combined (sensible- and latent) thermal storage materials that can store and dissipate energy in the form of heat [21]. In PCMs, heat is absorbed and released when the material changes its state from solid to liquid and vice versa. This is schematically shown in Figure 1.

During the heating pass of the phase change temperature, PCMs change from solid to liquid and capture heat, thereby reducing the temperature rise in the system. During the cooling pass of the phase change temperature, PCMs change from liquid to solid and release the stored energy, thereby reducing the cooling rate of the system. Therefore, PCMs can be used in concrete technology to reduce cracking caused by temperature effects [23]: early age cracking due to hydration of large concrete sections, and freeze/thaw damage in cold climates.

This paper aims to review the current state of the art of using PCMs for smart crack control in reinforced concrete, with the focus on the two aforementioned causes of cracking. The paper is organized as follows: first, different types of phase change materials and possible ways of application in concrete technology are discussed; then, studies regarding the use of PCMs to reduce cracking are reviewed; further, the influence of PCM addition on the properties of concrete is discussed; this is followed by a review of modelling approaches for PCMs in concrete; finally, conclusions and possible directions for future research are given.

## 2. Phase Change Materials in Concrete Technology

### 2.1. Classification of Phase Change Materials

**Figure 2:**
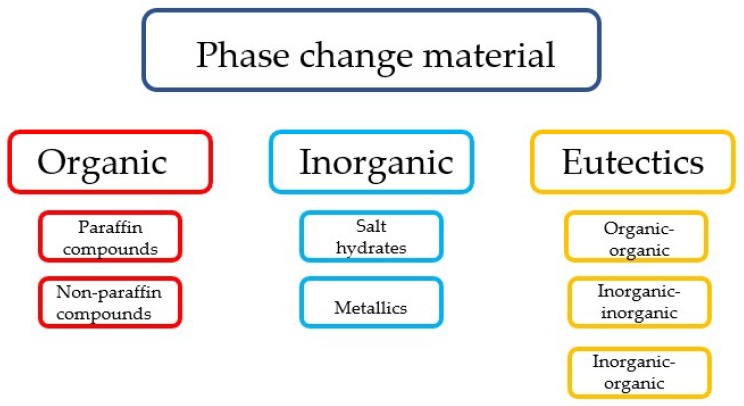
Classification of phase change materials (PCMs) (adapted from [21]).

A large variety of PCMs are available. In general, they can be divided in three categories (Figure 2): organic, inorganic, and eutectic PCMs. Organic PCMs are classified as paraffin and non-paraffin compounds [21,24,25]. Most organic PCM compounds are chemically stable, safe, and non-reactive [25]. Furthermore, organic PCMs in general do not suffer from phase segregation and crystallize with little or no supercooling [26]. Organic PCMs can be divided into two categories: paraffin compounds and non-paraffin compounds (such as fatty acids). Inorganic PCMs are classified as salt hydrates and metallics [21,24,25]. Inorganic PCMs may have potential applications in some types of building materials, because of their high volumetric heat storage capacity and good thermal conductivity [25]. In addition, they are relatively low-cost, readily available, and non-flammable [25]. However, most inorganic PCMs are corrosive to metals and undergo supercooling and phase decomposition [24]. A eutectic is a minimum-melting composition of two or more components, each of which melts and freezes congruently forming a mixture of the component crystals during crystallization [21]. This allows the design of organic/inorganic PCM mixtures to create optimum operating temperatures for specific applications [26]. Table 1 presents a comparison of the advantages and disadvantages of the three types of PCMs.

### 2.2. Incorporation of PCMs in Concrete

Most applications of phase change materials in construction are related to increasing the energy efficiency of the building envelope [24,25,26]. These applications have been a subject of numerous excellent reviews, and therefore are not the topic here. However, various techniques for incorporation of PCMs in building materials have been developed in these applications. The same techniques can be used when PCMs are utilized to control temperature variations within the material itself. The main approaches are:

- Using pipes filled with PCM incorporated in concrete.
- Using porous carriers such as lightweight aggregates (LWAs) impregnated with PCMs.
- Using microencapsulated PCMs.
- Impregnating PCMs in the concrete pores from the surface.

**Figure 3:**
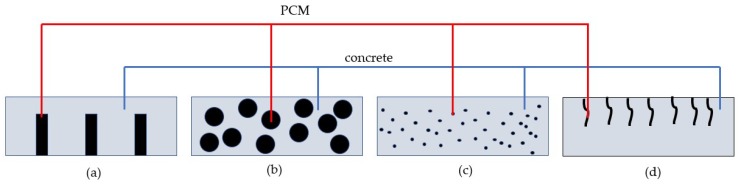
Methods for PCM incorporation in concrete: (**a**) using pipes filled with PCM; (**b**) using lightweight aggregate particles impregnated with PCM; (**c**) using microcapsules with PCM; (**d**) filling concrete surface voids via PCM absorption (adapted from [29]).

These methods are schematically illustrated in Figure 3.

#### 2.2.1. Embedded Pipes

For reducing temperature effects in massive concrete structures, the use of cooling pipes with running water is relatively common [30]. In order to increase the heat capacity of the embedded pipe systems, it has been suggested to fill them with PCMs with an appropriate phase change temperature. In general, a closed pipe system is used [29,31,32,33]. However, one study used a running system where water was replaced with PCM which was replaced every time a predetermined temperature was achieved, thereby increasing the rate of heat removal from the system [34]. A major advantage of the pipe system is that it is resistant to physical and chemical damage that it may undergo during casting and exploitation of concrete. Furthermore, leakage of PCM and its possible influence on fresh and hardened concrete properties are avoided when the pipe system is used. On the other hand, this approach has several drawbacks: first, the effect of the PCM is limited by the spacing of the pipe system; second, it is possible that the PCMs melt only partially due to their low thermal conductivity, resulting in an underutilized system; and finally, the system is as complicated as current cooling pipe systems.

#### 2.2.2. Lightweight Aggregates

**Figure 4:**
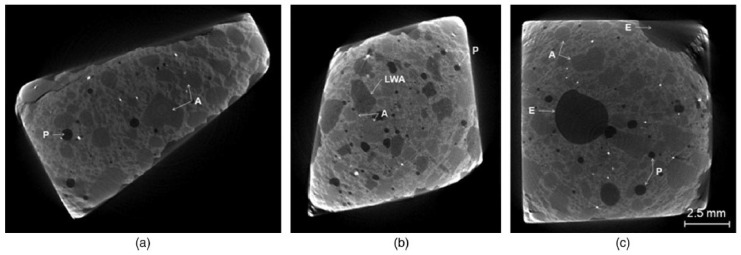
Microtomographs of investigated mortars: (**a**) control mortar without PCM, in which quartz aggregate (A) and pores/air voids (P) are easily visible; (**b**) mortar containing lightweight aggregate (LWA)-PCM, which appears similar to quartz aggregate but darker because of its lower density; and (**c**) mortar containing encapsulated PCM pellets (E) [43].

Another way of incorporating PCMs in concrete is through impregnation in lightweight aggregates. Water impregnated LWAs are commonly used for internal curing of concrete [35,36,37], providing additional water for hydration of high-strength concrete and thereby reducing its autogenous shrinkage. LWAs impregnated with bacteria and nutrients are also used in self-healing concrete [38,39]. Using the same approach, LWAs can be impregnated with suitable phase change materials, and then used in concrete (Figure 4).

**Figure 5:**
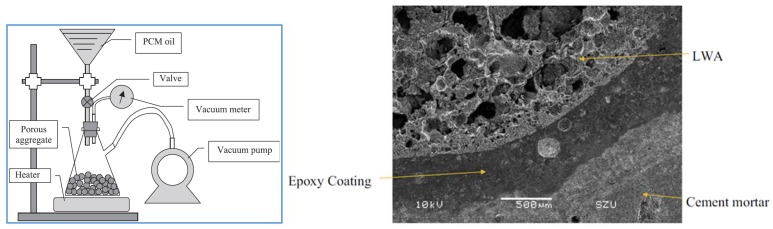
(**left**) Schematics of a vacuum impregnation procedure for LWA [40]; (**right**) scanning electron microscope (SEM) image of a PCM impregnated LWA particle coated with epoxy to prevent PCM leakage (scale bar given in the corner) [46].

Different types of LWAs have been used: expanded shale [23,29,32,40,41,42], expanded clay [40,43,44,45,46], perlite [42,47,48], and others such as diatomite and vermiculite [49,50,51,52]. These LWAs are impregnated with (liquid) PCMs using either immersion/direct impregnation (e.g., [23,42,43,44,45,49]) or a vacuum impregnation procedure (e.g., [29,32,46,47,48]). Schematic representation of the vacuum impregnation procedure is given in Figure 5. Both of these procedures have advantages and disadvantages. While vacuum impregnation enables higher levels of saturation with PCM (see e.g., [47] for comparison), soaking under ambient conditions is easier to replicate in practice [42]. It has to be noted that the PCM absorption capacity of the LWA cannot be simply determined by water absorption measurements: due to the low viscosity of water, it is able to penetrate into the pore spaces of smaller diameter and hence occupy more pore space than the PCM [46]. Furthermore, using only porosity is also not useful in this respect: a combined mercury intrusion porosimetry (MIP) and PCM absorption study of Aguayo [42] showed that porous aggregates with larger pore sizes of comparable porosities have larger PCM absorption. In fact, for a rough estimation of the penetrating degree of liquid PCM into LWA, the cumulative pore volume curve needs to be known: Zhang et al. [40] estimated that the liquid PCM can penetrate pores larger than 1–2 µm under vacuum condition. Clearly, a larger threshold value holds if LWAs are immersed in a PCM solution under atmospheric pressure.

Compared to the method of embedded pipes, use of LWAs as PCM carriers provides a better spatial distribution of PCM. Especially in composites with relatively fine LWA the PCM will be well distributed, and the ability of heat to travel through the matrix and reach all corners of the system is not as important [44]. Essentially, thermal diffusivity of the cement matrix must be balanced by the spatial distribution of the PCM, similar to the concept of ‘‘protected paste volume’’ in internal curing studies [36]. When PCMs are less homogeneously distributed (such as the case when embedded pipes are used), increasing the thermal conductivity of the composite is important [43]. In some studies wherein coarse LWAs are used, this is addressed by utilizing graphite powder [46], multi-wall carbon nanotubes [50], or aluminum powder [53] which increase the thermal conductivity of the LWA/PCM particles.

Another major disadvantage of the LWA approach is possible leakage of the PCM during mixing and/or exploitation and its dissolution in the mixing water. If the ambient temperature is lower than the phase change temperature of the PCM, leakage during mixing will occur [47]. In fact, some PCMs such as polyethylene glycol (PEG) are known to retard cement hydration [23]. Furthermore, if the LWA exchanges PCM with water from the matrix, it will dehydrate the system and result in a lower degree of hydration [43]. It has been also suggested that PCMs could in these cases coat the cement particles, protecting them from reacting with water [43]. One study in which methyl laureate PCM impregnated in LWA was used showed that, in certain cases, an expansive chemical reaction can occur between the PCM and aluminate phases in the cementitious matrix, causing cracking [29]. On the other hand, PCM in surface pores of the LWA could impair the LWA/cement paste bond, thereby reducing the strength of the composite material [45]. In order to mitigate these issues, some authors suggested coating the PCM impregnated LWAs: Memon et al. [46] coated the particles with an epoxy resin (Figure 5), while Ramakrishnan et al. [47] used a hydrophobic agent. Although coating of the LWAs will ensure that no leakage occurs, it will increase the complexity and the price of the production process.

#### 2.2.3. Microencapsulation

Microencapsulation is defined as a process in which tiny particles or droplets are surrounded by a coating to give small capsules with useful properties. In concrete technology, microencapsulation has been used for carrying self-healing agents [54,55,56] and corrosion inhibitors [57]. The primary advantage of microencapsulated PCMs are their chemically-inert nature (due to the polymer barrier between the PCM and any other material), the optimized heat transfer due to a high surface area-to-volume ratio, and their ability to be readily mixed into or coated onto other materials [58]. The shell needs to fulfil two major conditions: first, it needs to be able to sustain mixing and casting of concrete without damage and consequent leakage of the core material; and second, it needs to have long term stability in the highly alkaline environment in concrete.

**Figure 6:**
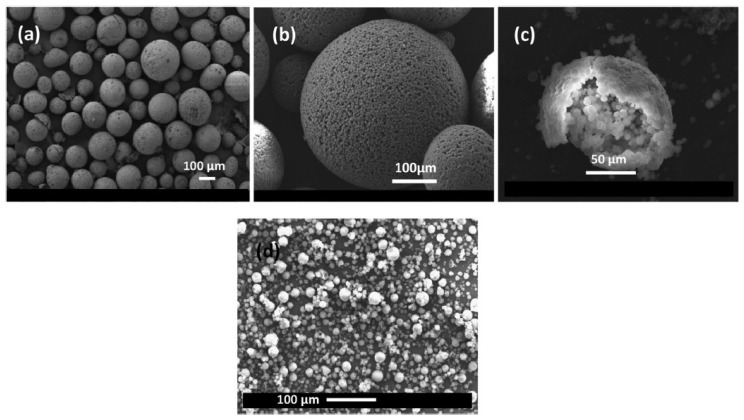
Micrographs of two types of PCM microcapsules: (**a**) PCM-A; (**b**) and (**c**) PCM-A showing smaller capsules that are agglomerated to form the larger capsule, and (**d**) PCM-B, which is composed of discrete particles [65].

**Figure 7:**
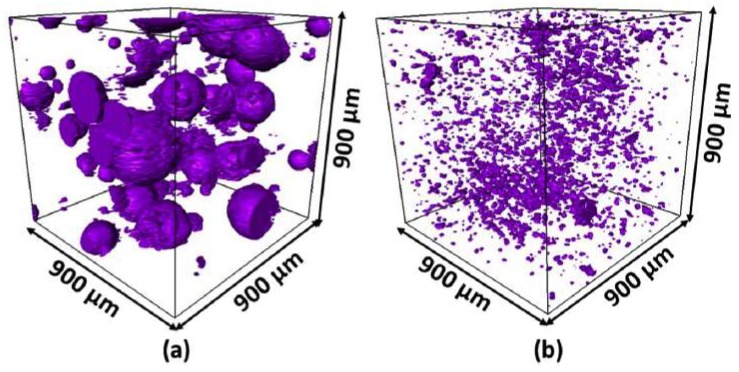
X-ray computed tomography scans showing distribution of two types of PCM microcapsules in cement paste: (**a**) PCM-A (agglomerated microcapsules, see Figure 6); and (**b**) PCM-B (discrete particles, see Figure 6) [65].

In most studies, paraffin as the PCM core material is encapsulated in a polymeric shell. Most microencapsulated PCMs used in concrete have either a polymethyl methacrylate (PMMA) [59,60,61,62,63,64,65] or melamine formaldehyde (MF) shell [63,66,67,68,69,70]. In one study, urea formaldehyde was used as a shell material [71]. Typically, PCM microcapsules are relatively small, in the size range of 10s of µm (Figure 6). However, microcapsules in the size range of 200–500 µm have also been used [71]. The small particle size is optimal in terms of surface to volume ratio of the PCMs due to their low thermal conductivity. Furthermore, small particles can be better dispersed in the cement matrix compared to large or agglomerated particles (Figure 7).

A major concern when using microencapsulated PCMs in concrete is their survivability and long-term durability. Survivability can be defined as the ability of microcapsules to survive the mixing process of concrete without rupture and leakage of the core material. Since this is a major concern, in most studies care is taken to minimize the exposure of the microcapsules to concrete mixing, for example by adding them as last ingredients [72,73,74]. Nevertheless, several studies have shown that a certain percentage of microcapsules does break during the mixing. For example, a large number of broken microcapsules were observed by Hunger et al. [72]. Other studies have also reported that a certain percentage of PCM microcapsules has ruptured during mixing [22,59,61].

**Figure 8:**
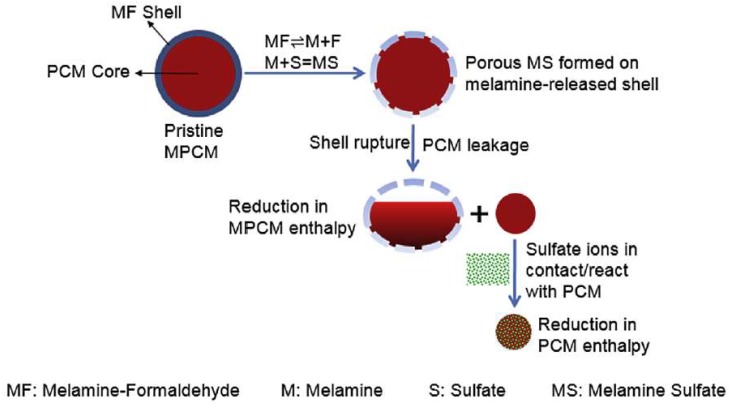
The proposed chemical interaction pathway which results in enthalpy reduction of the PCMs following exposure to caustic solutions containing sulfate ions [69].

On the other hand, in some studies it was emphasized that the microcapsules can survive the mixing process [63,69,74]. The issue then becomes their durability: the ability of microcapsules to maintain their integrity in the alkaline environment of the concrete pore solution while subjected to (mechanical) loading. Wei et al. [69] observed a 25% reduction in enthalpy of the phase change of PCM microcapsules regardless of whether mechanical mixing was performed or not. This was attributed to a chemical reaction of the melamine formaldehyde shell with sulfate ions, causing the release of the core material and its reaction with the pore solution. The proposed mechanism is schematically described in Figure 8.

**Figure 9:**
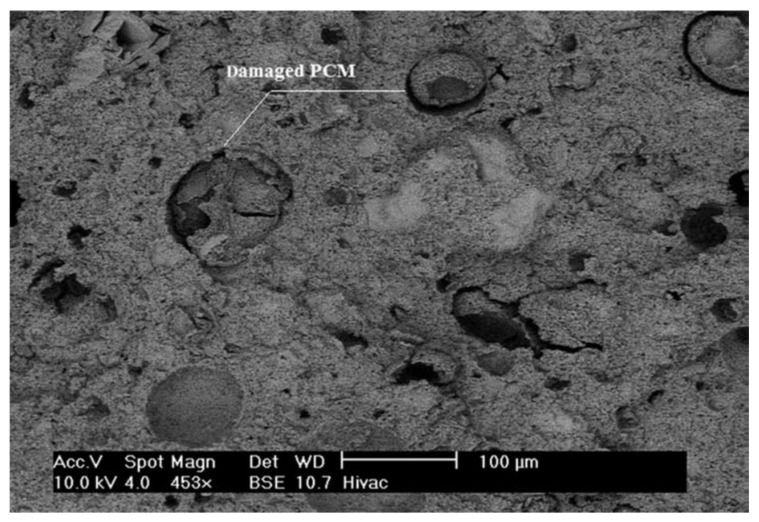
SEM micrograph (using back-scattered electron (BSE) detector) of concrete fracture surface showing damaged or collapsed PCM particles still occupying their original void [75].

**Figure 10:**
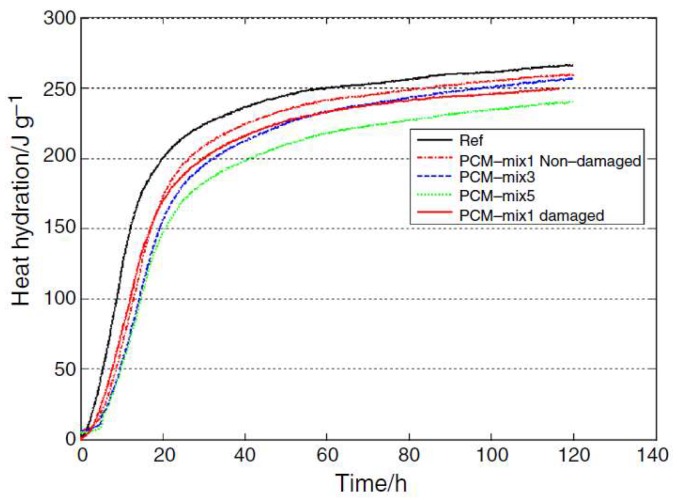
Heat release curves and comparison between damaged and non-damaged cases, showing lower hydration heat in the case when microcapsules are damaged [59].

Another possible cause of capsule rupture post-curing is mechanical loading. For example, Jayalath et al. [74] observed capsules that ruptured during cracking of the concrete. They suggested that the polymeric shell of the capsule is a soft material compared to the cementitious matrix. Consequently, the microcapsules act as crack initiation sites during loading, and the fracture propagates across the weakest point in the matrix, and this includes the PCM particle (Figure 9). This was also observed by others [73,75]. This is also in accordance with a study performed on a model gypsum plaster material containing controlled spherical porosity that acted as the initiation point for fracture [76]. Irrespective of the cause of the rupture, leakage of the core material may have consequences for the behavior of the composite. As already stated, it can reduce the enthalpy of the phase change, thereby reducing the thermal efficiency of the material [59,61,69]. Furthermore, if the leakage occurs during mixing, it can influence the cement hydration process. Eddhahak et al. [59] studied the effect of PCM leakage on the hydration process by intentionally damaging the capsules prior to mixing of the concrete. In their study, mortars with damaged PCMs exhibited a slight decrease in hydration heat (Figure 10) that was attributed to paraffin leakage that could have influenced the hydration process. Therefore, care should be taken when estimating thermal properties of cementitious materials with PCM microcapsules: if capsule breakage is neglected, material efficiency may be overestimated compared to reality.

#### 2.2.4. Surface Impregnation

Wallboards with surface impregnated PCMs have been proposed for latent heat storage in buildings [77,78,79]. Furthermore, concrete blocks impregnated with PCMs were also produced [77]. However, since PCMs incorporated in this way are susceptible to leakage, surface impregnation has so far not been used for control of temperature related cracking in cementitious materials.

## 3. Use of PCM to Reduce Concrete Cracking

### 3.1. Cracking of Concrete at Early Age Due to Hydration Heat Development

The temperature in young concrete increases due to the exothermic nature of the chemical reactions occurring during cement hydration [80]. If a concrete element is not restrained, it will freely expand during heating and contract during subsequent cooling, without inducing stresses [81]. In practice, however, concrete is almost always restrained to a certain degree, either by adjoining structures (external restraint) or internally due to a temperature gradient in the structural element itself (internal restraint). Massive structures, such as dams, are prone to early age thermal cracking [82,83,84]. The concrete surface will cool down faster than the core, giving rise to a temperature gradient between different layers of the structure/element. Differences in thermal dilation between parts of the structure will cause occurrence of tensile stresses at the concrete surface; if these stresses are higher than the tensile strength of concrete, cracking will result [85,86]. Thermal cracking will depend on material, structural, and execution factors [87]. Common measures used in practice for tackling this problem include: (1) changes in concrete mix design (using blended cements or lower cement content); (2) modifications in structural design (such as additional reinforcement, pre-stressing, expansion joints); (3) adapting execution parameters (by using cold mix ingredients or built-in cooling pipes). Clearly, use of phase change materials with an appropriate phase change temperature could be of great use in reducing the risk of thermal cracking in young concrete [23].

The first study proposing use of PCMs for temperature control in early-age concrete was performed by Mihashi et al. [88], who added paraffin microcapsules to the mixture. Their study showed that the maximum achieved temperatures in semi-adiabatic conditions were significantly lower when PCMs were incorporated in the mix.

Two methods of incorporating PCM have been used for crack control in young concrete: using pipes filled with PCM and using PCM microcapsules (see Figure 3). Qian et al. [33] incorporated PCM (sodium sulfate decahydrate with a phase change temperature of 32.4 °C and a heat of fusion of 241 J/g) in pipes cast in concrete, which was left to hydrate in semi-adiabatic conditions. Varying quantities of PCM (0%, 3%, and 6% of cement mass) were incorporated in concrete. They showed that pipes with PCM can clearly reduce the peak temperature in semi-adiabatic conditions. Furthermore, they also delay the onset of the peak temperature.

**Figure 11:**
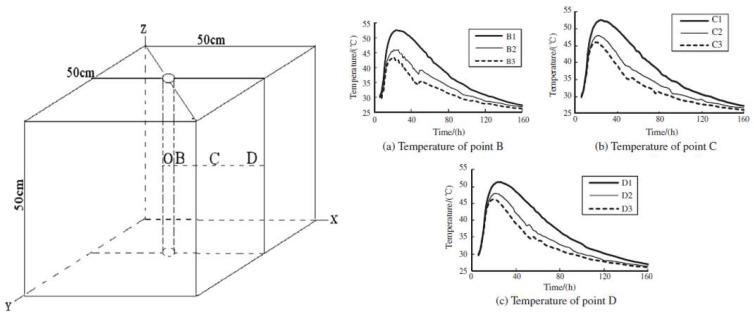
(**left**) Specimen geometry and points where temperature was measured; (**right**) temperature evolution in points B, C, and D (numbers 1, 2, and 3 mark cases where no cooling was used, water cooling was used, and PCM cooling was used, respectively) [34].

In another study, Qian & Gao [34] used an open circuit system: they replaced the water in the cooling system with a PCM suspension (Figure 11 left). The PCM suspension was replaced with a fresh one every time its temperature reached 25 °C. This enabled a significantly higher capacity to capture heat compared to a stationary PCM system. Furthermore, it was shown to be more efficient than using water as a cooling agent (Figure 11 right). Comparing to concrete with no cooling measure used, the temperature peak of concrete cooled by water and PCM suspension can be decreased to 76.1% and 84.9%, respectively. Furthermore, the temperature gradient around the cooling pipe was less steep in the case where PCM suspension was used as a cooling liquid instead of water. Therefore, they concluded that such a system could additionally reduce the probability of temperature induced cracking.

**Figure 12:**
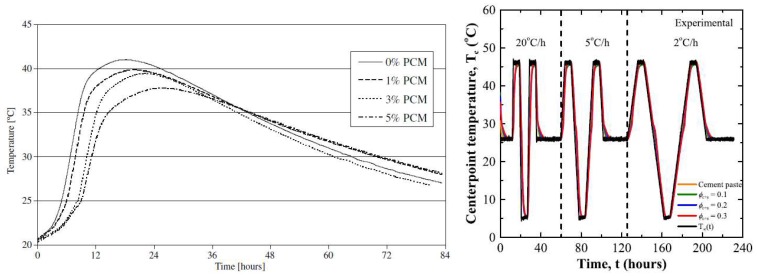
(**left**) Temperature development of four self-compacting mixes in a semi-adiabatic environment during the first 3.5 days after casting [72]; (**right**) Center point temperature as a function of time within cement paste specimens without and with microencapsulated PCM (phase change temperature 32 °C) with a volume fraction of 0.1, 0.2, or 0.3 subjected to an imposed chamber temperature T∞(t) varying at a ramp rate of 20, 5, and 2 °C/h [89].

More commonly, microencapsulated PCMs have been used for temperature control in young concrete [22,63,73,89,90]. Hunger et al. [72] used commercially available PCMs with a phase change temperature of 23 °C to reduce the temperature rise in hardening self-compacting concrete. They clearly showed that incorporation of microencapsulated PCM in the mixture caused a reduction in peak temperature in a semi-adiabatic test (Figure 12 left). Furthermore, a delay in the occurrence of maximum temperature was observed. The effectiveness of PCM microcapsule addition is proportional to the amount of PCM addition: the higher the PCM percentage, the lower the maximum temperature achieved and the later it occurs. Fernandes et al. [22] observed that, although there is a decrease in maximum temperature, the rate of temperature rise remains similar to the reference in mixtures incorporating PCMs. Snoeck et al. [63] showed that the effectiveness of PCMs in reducing the maximum temperature depends on the phase change temperature. In their study, when a PCM with a phase change temperature of 18 °C was used, there was only a minor effect since the phase change temperature was below the initial testing temperature (20 °C). On the other hand, when the PCM with a phase change temperature of 28 °C was used, the effect of encapsulated PCM could only start when the cement hydration reaction was already at “full speed”, so the effectiveness was less pronounced. Their study stresses an important fact: the phase change temperature needs to be adjusted for different climatic conditions in order to be fully utilized. This was further addressed by Young et al. [90], who used a PCM with a phase change temperature of 40 °C in the warm climate in California, USA. It is also important to notice that the incorporation of microencapsulated PCMs reduces the rate of cooling, which could be even more critical for controlling early age cracking in concrete [22].

In addition, one study (by Kim et al. [28]) used barium based PCM which was directly added to the concrete mix. The PCM based mix showed excellent performance, with a semi-adiabatic temperature rise lower than the mixtures incorporating supplementary cementitious materials, which are commonly used to combat early age temperature rise.

In warm climates, incorporation of PCM has an additional advantage that goes beyond the early age behavior. Since PCMs typically remain active, they will go through a phase change every time their phase change temperature is passed. Therefore, they can contribute to reducing thermal fatigue in structures exposed to temperature variations, such as concrete pavements. Thiele et al. [89] showed that the addition of PCM microcapsules is able to “smooth” the temperature cycles in concrete (Figure 12 right). It has to be noted that the rate of temperature change has a significant effect on the rate of temperature change (as also observed by Fernandes et al. [22]): if the rate of temperature change is too fast, the benefits of heat absorption/release are overwhelmed as the concrete temperature is already higher/lower than temperature of phase change. In such cases, the thermal response of the concrete is governed by the sensible instead of the latent heat, which cannot stabilize the concrete temperature.

### 3.2. Cracking of Concrete Due to Freeze/Thaw Cycles

In cold climates, concrete may deteriorate due to freezing and thawing of water in the system [80,91]. Since the volume of ice is around 9% higher than the volume of water, expansive pressures are present in the concrete when freezing temperatures occur if there is not enough space to accommodate this expansion. If the water saturation level is higher than the critical value (theoretically 91%, but in practice around 86%−88% [92]), damage may occur [93,94,95,96]. In practice, this is solved by air-entrainment, whereby air bubbles are intentionally introduced in the concrete by means of chemical additives [97]. If they are well distributed and properly spaced, they are able to accommodate the volumetric expansion without causing pressure or cracking [98].

The described approach essentially tries to avoid damage by accommodating the expansion. An alternative would be to avoid the cycles of freezing and thawing altogether. In the past few years, several studies have proposed the use of phase change materials to reduce the number of freeze/thaw cycles and improve concrete durability. The use of phase change materials in concrete technology for reducing freeze/thaw damage was first proposed by Bentz and Turpin in 2007 [23]. In this work, they performed numerical simulations in order to quantify the effect of PCM incorporation in concrete on the number of freeze/thaw cycles in twelve locations across the US, spanning a range of climates. They assumed that concrete contains 300 kg/m^3^ of PCM with a phase change temperature of 5 °C and the enthalpy of phase change of 250 J/g. Since previous works showed that a greater number of freeze/thaw cycles can be expected in bridge decks compared to concrete pavements [99], a bridge deck was simulated. They concluded that the hypothetical concrete with PCM could reduce the number of freeze/thaw cycles by 30% on average across selected geographic locations in the US. However, they stated that the efficiency of PCMs will be strongly climate dependent—the ideal situation being a climate with numerous freeze/thaw cycles but without extremely cold weather.

**Figure 13:**
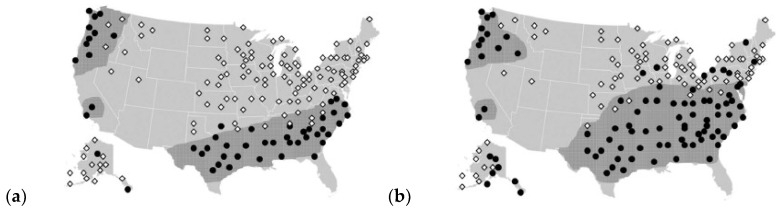
(**a**) US locations in which incorporation of 50 kg/m^3^ of PCM increases bridge deck service life by less than 1 year (white diamond) or more than 1 year (black dot); and (**b**) US locations in which incorporation of the maximum 120 kg/m^3^ of PCM increases bridge deck service life by less than 1 year (white diamond) or more than 1 year (black dot); shaded areas indicate a rough estimate of the regions in which PCM incorporation is practical for extending bridge deck service lives [43].

Based on this, an additional study was performed by Sakulich and Bentz [43]. In the study, they performed more advanced simulations of freezing and thawing for various climates in the US (Figure 13). They concluded that the optimal range of phase change temperatures of PCMs for preventing freeze/thaw damage is between 3.5–6 °C. This is because of the way bodies are cooled, which depends heavily on the thermal conductivity of the system. Simulations of Sakulich and Bentz [43] show that increasing the thermal conductivity of the system will result in fewer freeze/thaw cycles if the phase change temperature is in the optimal range. Cooling of a hot body will occur primarily due to convection or conduction. A greater thermal differential will lead to a more rapid cooling; a greater thermal conductivity will lead to a more rapid transfer of heat from the interior of the body to the surface and even cooling. In cases when thermal conductivity is low, heat cannot flow from the interior to the surface easily, so although the surface may cool rapidly, the interior will be maintained at a higher temperature and remain free of freeze/thaw damage. Therefore, in such applications, increasing the thermal conductivity of the composite will be important [43]. This is especially an issue when LWAs with PCM are used (see Section 2.2.2.).

**Figure 14:**
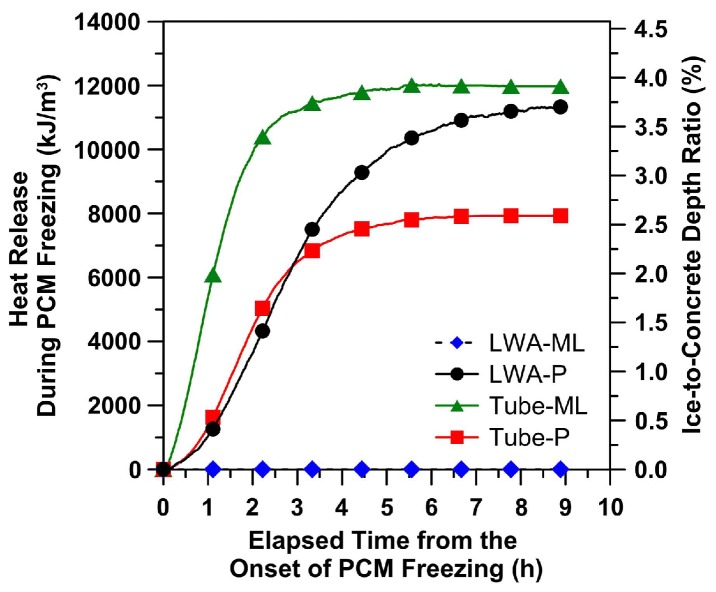
Released heat during PCM freezing in mortar specimen as a function of elapsed time from the onset of PCM freezing (notice that the quantity of PCM per volume of mortar sample varies for each case [29].

An additional benefit of using PCMs instead of air entrainment in this case is the ability of the material to “melt” ice and snow forming on its surface: because the concrete will remain warmer for a longer period of time, it will prevent snow from accumulating and ice from forming on the surface. This may be very beneficial for some applications, such as airport runway pavements. This possibility was explored in several studies. Farnam et al. [29] used two types of PCM (methyl laureate with a phase change temperature of 1.9 °C and latent heat of fusion of 160.4 J/g; and paraffin oil with a phase change temperature of 2.9 °C and latent heat of fusion of 129.4 J/g) either embedded in a tube system or incorporated in LWAs for melting snow on the concrete surface. Based on the energy released during the phase change and the volume of the concrete, they calculated the amount of ice that can be melted during the phase transition. In addition, they calculated the amount of heat released during the PCM phase transformation per cubic meter of concrete from a Low-Temperature Differential Scanning Calorimetry experiment. Both quantities are shown in Figure 14. It can be noted that concrete with ML (methyl laureate) impregnated in LWAs showed no improved performance, since a chemical reaction occurred between the PCM and cement hydrates. With PCM in embedded tubes, ML is able to melt a significant amount of snow. On the other hand, paraffin does a good job irrespective of whether it is impregnated in LWAs or embedded in tubes. Since ML has a higher heat of fusion, however, it performs the best in the case when it is embedded in a pipe system.

**Figure 15:**
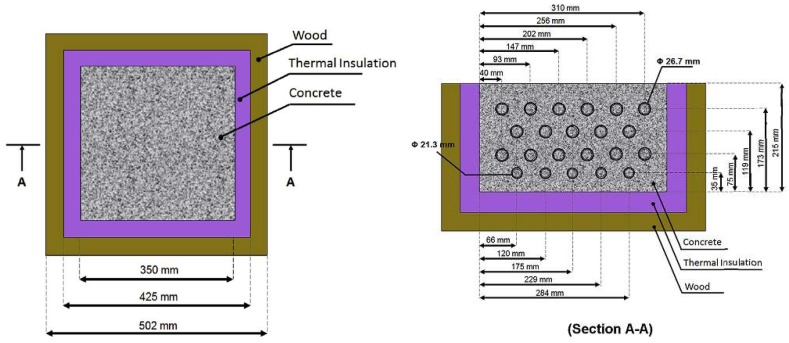
Design details for the large-scale concrete slab: (**left**) top view details of the mold and the concrete slab; (**right**) cross section details of the mold and the concrete slab with embedded pipes (to prevent heat transfer through metal pipes, additional outer thermal insulations were installed on both sides of the framework where pipes were out of the wood framework) [32].

**Figure 16:**
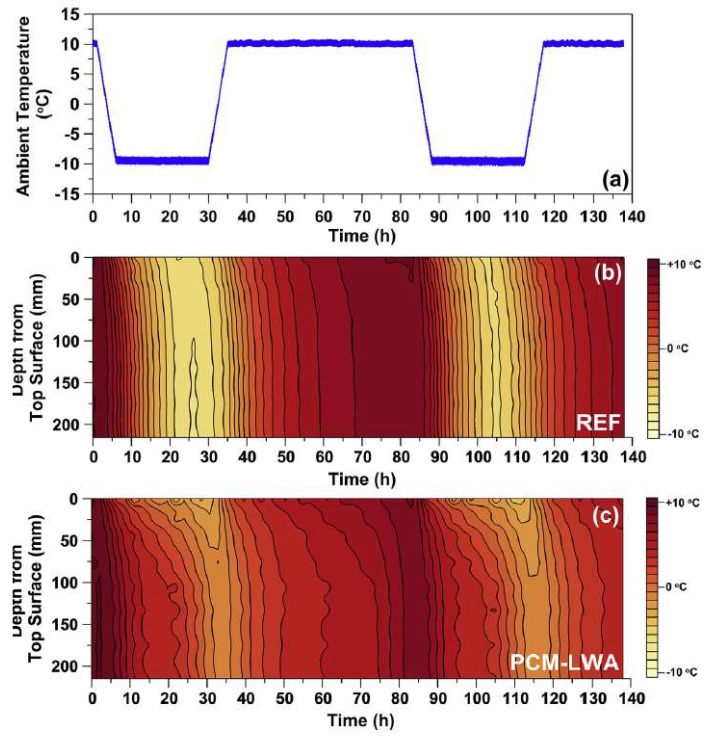
Thermal cycling of the concrete slab (**a**) ambient temperature applied in thermal cycling experiment; (**b**) temperature profile within the depth of reference slab as a function of time; and (**c**) temperature profile within the depth of PCM–LWA as a function of time [32].

Based on these results, Farnam et al. [32] performed a follow-up study. In it, they prepared large scale slabs with (LWA and embedded tubes) and without PCMs. Their experimental setup is shown in Figure 15. In the two approaches, there is a slight difference in the amount of PCMs used: in the LWA system, there was 22% of PCM per volume, while in the embedded pipe system, there was 14% of PCM per volume of the specimen. Specimens were insulated on the sides in order to ensure 1D heat transfer. The slabs were then subjected to temperature cycling with and without (manmade) snow. An example of cycles and temperatures within the slabs is given in Figure 16. Their research showed that both methods (i.e., PCMs impregnated in LWAs and embedded pipes) are able to melt snow at the concrete surface. However, some differences between the two approaches have been reported: when the concrete slab was exposed to an ambient temperature above the freezing temperature of PCM before a snow event, a relatively rapid heat release was observed for the embedded pipe method during PCM phase transformation, while the heat release in the LWA method was more gradual. The rapid heat release in the PCM-PIPE slabs may be beneficial in melting ice and snow at a faster rate than the PCM-LWA slabs; however, the capability of snow melting in the PCM-PIPE slabs may noticeably decrease when the concrete slab is exposed to an ambient temperature near or below the freezing temperature of PCM before a snow event. Conversely, for the LWA method, the gradual heat release can be used over a wide range of temperature variation and the snow melting capability remains relatively beneficial.

**Figure 17:**
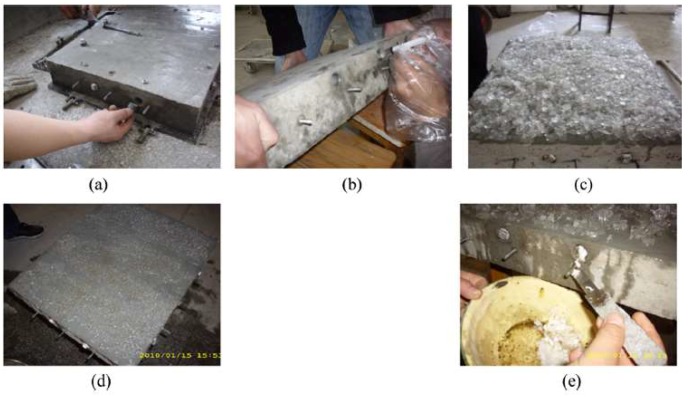
The simulation test is carried out under low temperature. (**a**) Injecting entrance design of PCM; (**b**) injecting PCM; (**c**) simulation of surface ice-snow condition; (**d**) melting ice conditions of surface; (**e**) phase change completion of PCM [31].

Gao et al. [31] developed a composite bridge deck with an anti-freezing capability. It contains a phase change functional layer which consists of embedded steel pipes filled with PCM with the aim of reducing the amount of ice and snow that forms, similar to [29,32]. Their study confirms that the use of PCM filled pipes is a good way of keeping structures such as bridge decks ice-free (Figure 17).

## 4. Effects of PCM on Concrete Properties

### 4.1. Fresh Concrete Properties

If PCMs are added to the mix as microcapsules or impregnated in LWAs, they may affect the properties of fresh concrete. If LWAs impregnated with PCMs are used, the following issue may occur: since PCMs are not able to penetrate into small pores of the LWA, these pores will remain air filled. If such LWA is placed in the mix, it may imbibe water from the concrete and thus dehydrate the system [100]. Sakulich and Bentz advised, therefore, that the LWAs be water saturated [43].

**Figure 18:**
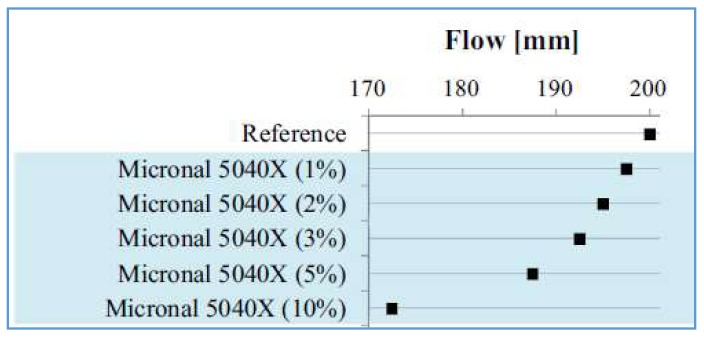
Flow test result for a mortar with a certain type of PCM microcapsules, showing a decrease in flow (workability) with increasing inclusion volume [63].

When microencapsulated PCMs are used, it has been shown that the workability of concrete may be affected due to their small size and, consequently, large surface area [62,63,64,72,101]. For example, Snoeck et al. [63] found that the addition of PCM microcapsules reduces the workability in proportional to the volume of PCM addition (Figure 18). The decrease in flow was observed irrespective of the way PCM microcapsules were added to the mix, i.e., by replacing a part of fine aggregates or by simply adding them to the mix. Due to these effects, Šavija et al. [73] needed a relatively high amount of superplasticizer for SHCC (strain hardening cementitious composite, an ultra-ductile cementitious material) mixes wherein large proportions of limestone powder were replaced by PCM. A drop in workability with PCM addition was also observed by Hunger et al. [72], but they were able to obtain concretes with good or partly excellent self-compacting properties.

### 4.2. Mechanical Properties

Addition of PCM to the concrete mixture may affect its mechanical properties. When it comes to compressive strength, two technologies of PCM incorporation (i.e., PCM impregnated LWAs and microencapsulation) result in different effects.

When LWAs are used to replace a part of the aggregates in the concrete mix, a drop in compressive strength is expected [44]. The same behavior is commonly observed for PCM impregnated LWAs [44]. However, this is caused by the weak nature of LWA particles. In fact, the compressive strength of the material will be similar irrespective of whether LWAs are impregnated with water or with PCMs [42], provided PCMs are contained inside the LWA only. It has been observed that the presence of PCM on the LWA surface may impair the aggregate-paste bond and so reduce the compressive strength [41,45]. It has also been observed that some PCMs leaking out of LWAs may chemically interact with hydration products causing expansion and cracking [29].

**Figure 19:**
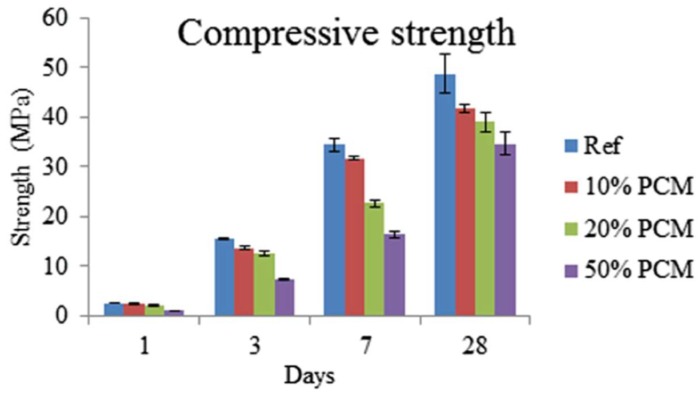
Compressive strength as a function of time for strain hardening cementitious composite (SHCC) mixtures containing different amounts of microencapsulated PCM (error bars indicate standard deviation) [73].

When microencapsulated PCMs are used, in general two opposing effects on concrete strength occur [74]: first, replacement of sand particles with softer PCMs causes a reduction in strength; second, small PCM particles act as nucleation sites for cement hydration, thereby contributing to strength increase [65]. A balance between these two effects will determine which one is dominant. In general, however, there is a drop in compressive strength of cementitious materials proportional to the PCM inclusion percentage (e.g., as shown in Figure 19) [22,60,62,63,68,72,73,75,102]. Therefore, there is a limit to how much PCM may be added in structural concrete.

It has been observed that, in general, the drop in tensile/flexural strength with microencapsulated PCM addition is significantly less pronounced than the drop in compressive strength [22,63,73,75,102]. However, the influences of PCM additions on the fracture properties (fracture toughness and critical crack tip opening displacement) are less substantial, especially so for lower PCM dosages. This is attributed to the beneficial effects of crack blunting and twisting which are expected to emerge on the addition of compliant inclusions [22]. Similar effects can be achieved through use of mineral additives such as silica fume and fly ash [103,104,105]. For crack control, this is more important than the compressive strength.

It should be noted that the strength of concrete with PCMs may be, to a certain extent, dependent on the temperature (i.e., below or above the phase change temperature of PCMs). Šavija et al. [102] found that the force needed to rupture the microcapsules themselves was higher for temperatures lower than the phase change temperature (i.e., when PCM is solid) than for temperatures above the phase change temperature (i.e., when PCM is liquid). Pilehvar et al. [106] observed a temperature dependence in the compressive strength of geopolymer concrete incorporating PCM microcapsules. However, this dependence was not observed in other studies, and therefore more research is needed in this field.

### 4.3. Thermal Properties

**Figure 20:**
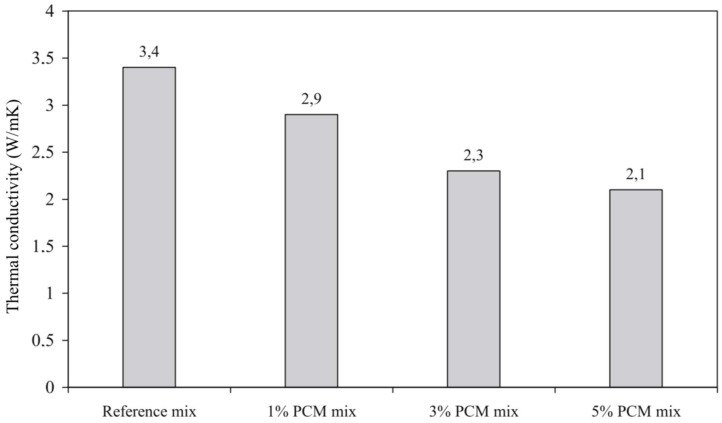
Thermal conductivity of the self-compacting concrete PCM mixes (weight% of PCM) [72].

Thermal conductivity of PCMs is relatively low compared to cementitious materials (Table 1). Therefore, it is expected that the thermal conductivity of PCM cementitious composites would be somewhat reduced. For concrete with PCM impregnated LWA, a reduction in thermal conductivity was reported in multiple studies [42,43,44,47]. Memon et al. [46] proposed adding graphite powder to increase thermal conductivity. When microencapsulated PCMs are added to the mix, thermal conductivity is found to decrease in proportion with the PCM addition [60,64,71,72,74,75] (e.g., Figure 20). Since this decrease may be significant, it needs to be taken into account when designing PCM cementitious systems and assessing their efficiency.

### 4.4. Durability

**Figure 21:**
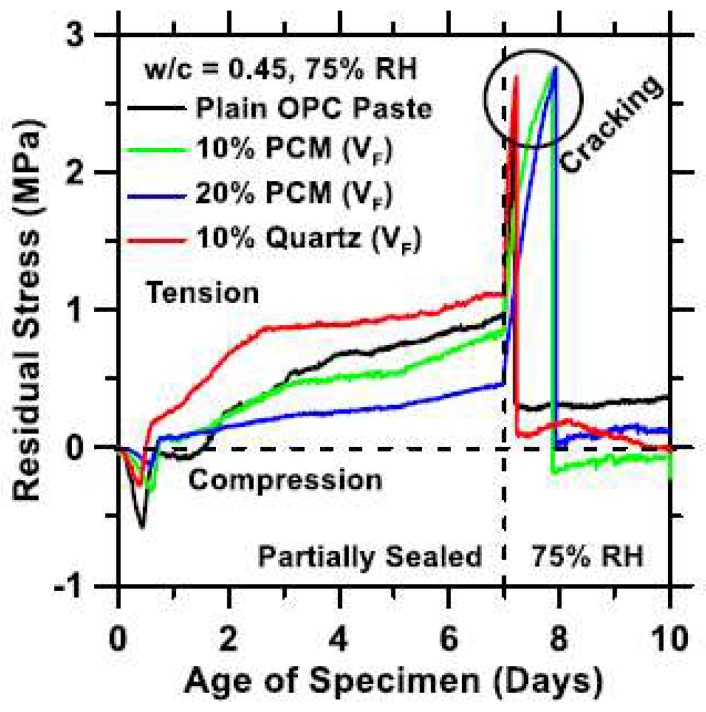
Residual stress development measured using the dual ring setup as a function of time for plain cement paste, and quartz and PCM inclusion dosed mixtures. After 7 days of partially sealed curing the specimens were dried symmetrically, i.e., from their top and bottom surfaces at 75% relative humidity (RH). The time at which the stress drops sharply indicates macroscopic damage localization (cracking), when a single-crack formed in the ring samples [68].

Durability of cement-based materials incorporating PCMs has been scarcely studied. When the long term behavior of such materials is considered, only shrinkage has received attention. It is expected that the addition of compliant inclusions, such as PCM microcapsules, into the mix will cause an increase in shrinkage deformation [80] compared to stiff inclusions (i.e., quartz sand). In fact, it has been observed that the drying shrinkage of cement paste with PCM microcapsules is higher than that of cement paste with the same volume of quartz sand [22]. This is because PCM microcapsules do not restrain paste shrinkage and fulfil a role similar to air voids (in the context of shrinkage) in the system [69]. In these cases, shrinkage is controlled by the paste response [22,68]. Shrinkage of cement pastes with PCM microcapsules is not different from shrinkage of plain cement paste. Based on this observation, and the fact that the strength of the material decreases with PCM addition, it would be expected that PCM-cement composites are more susceptible to restrained shrinkage cracking. In fact, it has been shown that they are more resistant (i.e., that cracking occurs at later age) compared to equivalent mortars with quartz sand [68] (Figure 21). This has been attributed to cracking blunting and deflection caused by soft PCM inclusions, and to a lesser extent to higher stress relaxation [68].

Transport properties of cement-based materials with PCMs have not been extensively studied. In the study by Wei et al. [69], it has been shown that the addition of PCM microcapsules reduces the water uptake of the material. Since the microcapsules are not porous, they act simply as inclusions in the matrix, increasing tortuosity and consequently slowing down the water transport. Such behavior is, in fact, expected, and has been observed in concrete as well [107,108,109]. Although other transport properties, such as chloride transport, have not been studied, it is expected that the addition of solid inclusions will have a similar effect on those [110,111].

The effect of PCM addition on reinforcement corrosion has been studied by Cellat et al. [112]. In their study, no influence of eutectic microencapsulated PCM on corrosion of reinforcement was observed. Since not many studies on this have been performed, more research is needed.

## 5. Modeling of PCM Composites

### 5.1. Effective Properties

As already described, properties that are most affected by the presence of PCM additions are (compressive) strength, elastic modulus, and thermal conductivity. Models developed to predict the effect of PCM addition on these properties are described.

#### 5.1.1. Mechanical Properties

**Figure 22:**
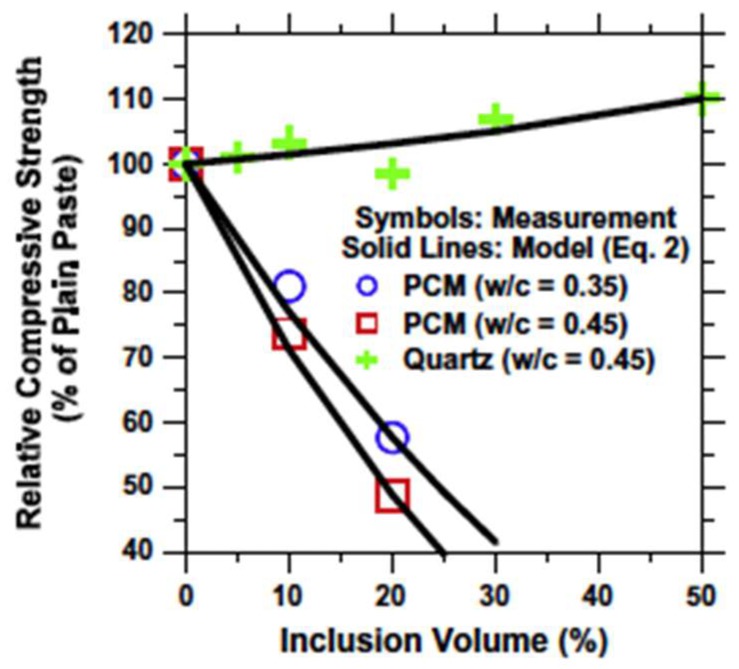
The relative compressive strength for mixtures containing different volume fractions of quartz and PCM inclusions [22].

As previously discussed, many studies reported a drop in compressive strength with PCM addition. Based on their experimental findings, Fernandes et al. [22] proposed the following relationship between the inclusion (PCM microcapsule) volume and the compressive strength [113]:

(1) $$\sigma_{n}=100{\left(1-\frac{V_{1}}{100}\right)}^{y}$$

where *σ_n_* is the compressive strength of any system normalized by the strength of the plain cement paste (unitless), *V_I_* is the volume fraction of inclusions in the system (%), and *y* is a fitting constant (unitless) that depends on the nature of the inclusion used. For microencapsulated PCMs and quartz sand used in their study, the following values of the fitting parameter y gave the best fit: *y* = 2.47 or 3.22 for PCM mixtures for w/c = 0.35 and w/c = 0.45, and *y* = −0.14 for the quartz mixtures for w/c = 0.45, respectively. A comparison between experiments and predictions is given in Figure 22.

**Figure 23:**
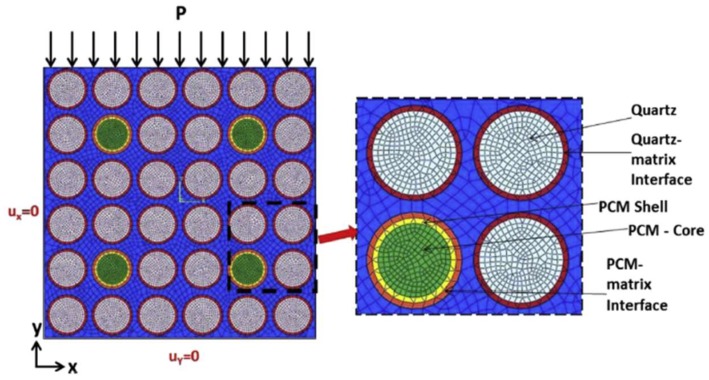
Finite element (FE) model showing the boundary conditions, applied compressive loading, and a magnified representation of the inclusions with the zones around them. The model contains 50% of inclusions (mortar with 10% PCM–E particles replacing quartz particles) by volume (or area) [65].

In the case when PCM inclusions are combined with stiff inclusions (sand), it is not possible to use analytical expressions to predict the strength. For this purpose, Aguayo et al. [65] developed a finite element model wherein inclusions such as sand particles and PCM microcapsules can be explicitly modeled (Figure 23).

The model showed that, when a combination of weak and soft inclusions (such as PCM microcapsules and quartz sand) is used, the influence of PCM addition on the compressive strength of mortars is more complex. The weaker paste-quartz interfacial elements resulted in the most likely failure path progressing around the inclusions. In systems where small amounts of quartz were replaced by PCM, localized stress concentrations were observed at the PCM-paste interface, but the PCMs being few and far between, the predicted failure region was still along the weaker paste-quartz interface. When the system contained a substantial amount of PCMs, the stress concentrations at the paste-PCM interfaces grew, and the likelihood of paste failure increased substantially. This was in accordance with their experiments [65], which showed somewhat higher compressive strength of systems with a small amount of PCM microcapsules compared to a reference OPC system. This is somewhat unexpected, because in a system where weak inclusions are added, a drop in strength proportional to the inclusion percentage is typically expected [76].

Similar approaches were proposed for determining the Young’s modulus of the composite. Fernandes et al. [22] proposed to use the following equation [114]:

(2) $$E_{C}=E_{p}(\frac{\left(1-2\nu \right)\left(1-V_{I}\right)}{\left(1-2\nu_{p}\right)\left(1+V_{I}\right)})$$

**Figure 24:**
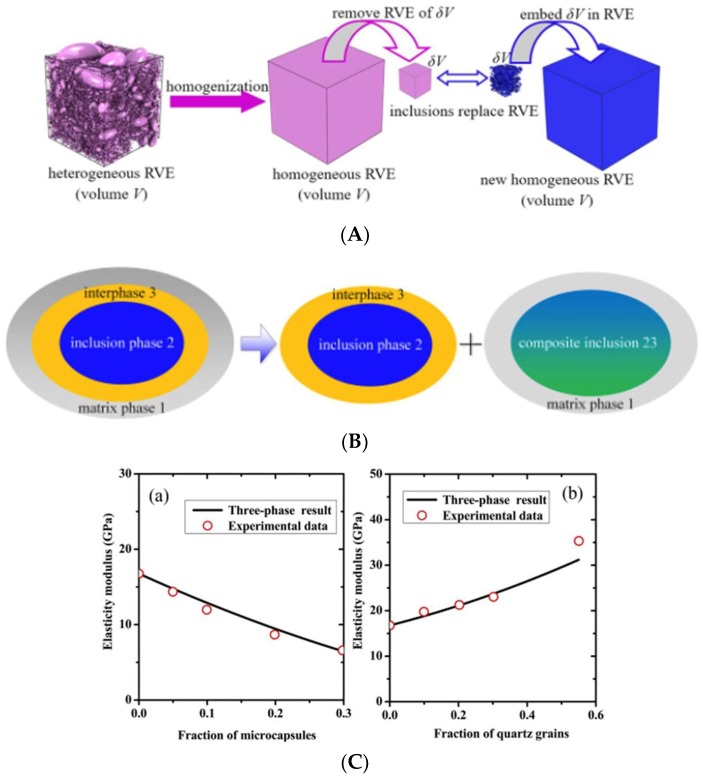
(**A**) Schematic view of incremental homogeneous of the differential effective medium (DEM) theory; (**B**) Schematic view of a three-phase representative volume element (RVE) divided into two two-phase cells. Aggregates (inclusion phase 2) and its adjacent interfacial transition zone (ITZ) (interphase 3) are mapped into a composite inclusion phase 23; (**C**) Comparisons of the predicted elastic moduli with the experimental data [115], (**a**) mortar containing microencapsulated PCMs; (**b**) mortar containing quartz inclusions.

Here, *E_C_* is the elastic modulus of the composite, *E_p_* the elastic modulus of plain cement paste, *ν* the Poisson’s ratio of the composite (determined by averaging the Reuss–Voigt solution in proportion to the volume fractions of paste and inclusions in the composite and their properties), *ν_p_* the Poisson’s ratio of the plain cement paste, and *V_I_* volume fraction of inclusions/PCMs (%). A more advanced approach based on micromechanics was proposed by Xu et al. [115]. In their framework, inclusions and their interfaces are considered explicitly (Figure 24). This way, systems containing both PCMs and quartz inclusions can be considered. The model showed a good agreement with experimental data.

#### 5.1.2. Thermal Conductivity

As already described, the addition of PCMs has a profound influence on the thermal properties of the composite material due to PCMs lower thermal conductivity. To simulate the performance of PCM based concrete in elements and structures, it is therefore necessary to predict the thermal conductivity of the composite material depending on its constituents.

For composites incorporating microencapsulated PCMs, Ricklefs et al. [67] proposed the use of an equation based on Felske’s model [116]:

(3) $$k_{eff}=\frac{2k_{m}\left(1-\phi_{C}-\phi_{S}\right)\left(3+2\frac{\phi_{S}}{\phi_{C}}+\frac{\phi_{S}}{\phi_{C}}\frac{k_{C}}{k_{S}}\right)+\left(1+2\phi_{C}+2\phi_{S}\right)\left(\left(3+\frac{\phi_{S}}{\phi_{C}}\right)k_{C}+2\frac{\phi_{S}k_{S}}{\phi_{C}}\right)}{\left(2+\phi_{C}+\phi_{S}\right)\left(3+2\frac{\phi_{S}}{\phi_{C}}+\frac{\phi_{S}k_{C}}{\phi_{C}k_{S}}\right)+\left(1-\phi_{C}-\phi_{S}\right)\left[\left(3+\frac{\phi_{S}}{\phi_{C}}\right)\frac{k_{C}}{k_{m}}+2\frac{\phi_{S}k_{S}}{\phi_{C}k_{C}}\right]}$$

**Figure 25:**
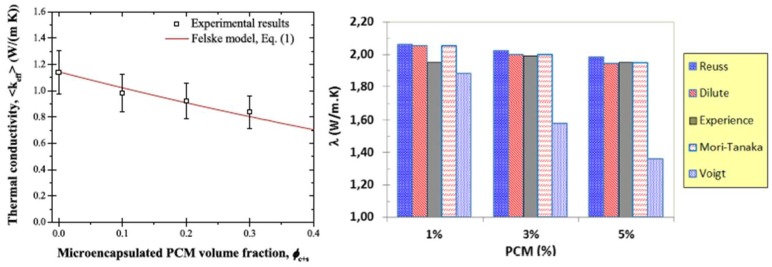
(**left**) Temperature-averaged effective thermal conductivity as a function of microencapsulated PCM addition, together with the predictions given by Equation (3) [67]; (**right**) Equivalent thermal conductivity calculated using four homogenization schemes, compared to experience [60].

Here, *k* and *ϕ* are the thermal conductivity and volume fraction and the subscripts *c*, *s*, and *m* refer to the core, shell, and matrix, respectively. This model showed very good agreement with experimental data (Figure 25). Eddhahak et al. [60] tested four different homogenization approaches for predicting the effective thermal conductivity of the composite: Voigt and Reuss bounds, dilute scheme, and Mori-Tanaka homogenization. Except for the Voigt model, other models showed a good match with experience (i.e., experimental data) (Figure 25).

**Figure 26:**
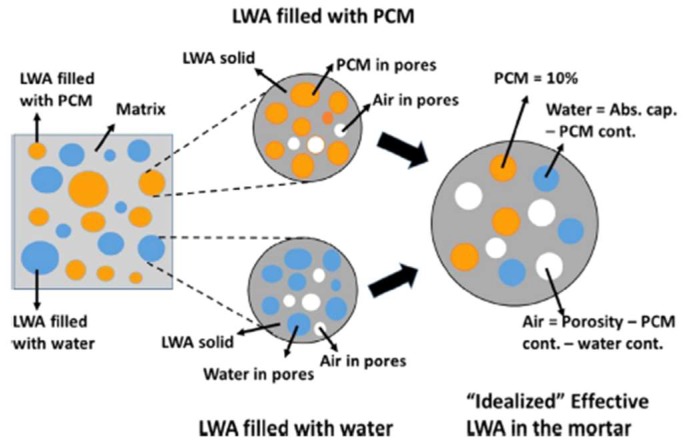
Idealized LWA for effective property determination. Some LWAs have PCM while some others have water to ensure desired PCM levels in the mortars. An ‘‘idealized” LWA is also shown, that is representative of the LWA phase [42].

The Mori–Tanaka homogenization scheme was also used by Aguayo et al. [42] to predict the thermal conductivity of concrete with LWA PCM. Their approach is outlined in Figure 26. According to Aguayo et al. [42], the application of Mori–Tanaka mean-field homogenization method that considered the known microstructural arrangement of the composite (i.e., a heterogeneous inclusion in a homogeneous matrix) resulted in a better predictive capability than the use of sequential homogenization based on thermal conductivity contrast of the different components in the composite.

**Figure 27:**
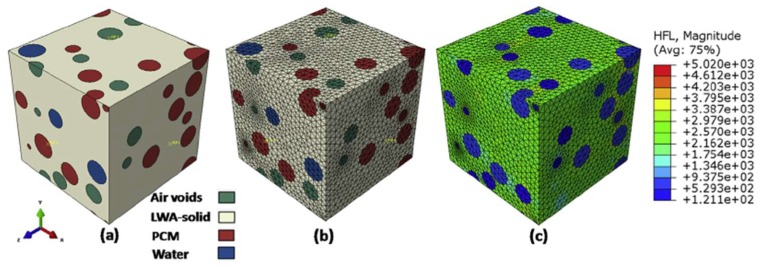
3D Microstructure-guided numerical process to determine the effective thermal conductivity of PCM-incorporated LWAs: (**a**) FE model showing the water-filled, PCM-filled, and air-filled pores in the LWA inclusions (Pumice); (**b**) FE mesh; and (**c**) heat flux distribution (W/mm^2^) under an imposed temperature difference of 15 °C [117].

**Figure 28:**
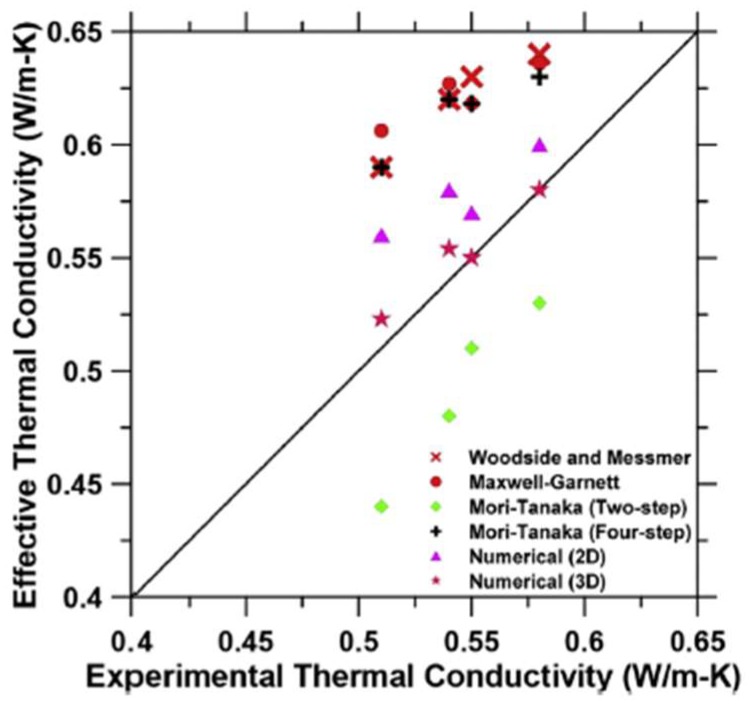
Predicted vs. experimental thermal conductivity of PCM-impregnated LWA mortars and comparison of different numerical and analytical techniques [117].

For more complex cases, and also 3-dimensional analyses, Das et al. [117] proposed the use of a microstructure-guided numerical model based on the finite element method. An example of a 3D microstructure with randomly distributed PCMs is shown in Figure 27. According to their analyses, three-dimensional numerical homogenization shows better agreement with the experiments compared to a 2D homogenization and several analytical homogenization approaches (Figure 28).

### 5.2. Material and Structural Performance

Time dependent heat transport can be described by the transient heat conduction equation for a stationary medium (in 1D) [118]:

(4) $$\rho c_{p}\frac{\partial T}{\partial t}=\frac{\partial }{\partial x}\left(k\frac{\partial T}{\partial x}\right)$$

Here, *ρ* is the density (kg/m^3^), *c_p_* the specific heat capacity (J/kg K), *k* the thermal conductivity (W/mK), *T* the temperature (K), *t* time (s), and *x* the spatial coordinate (m). In case of PCMs, the sensible heat contribution (proportional to the mass and the specific heat capacity of the material) and the latent heat contribution (proportional to the mass and the enthalpy of phase change) both need to be considered. In the literature, latent heat stored during the phase change in the PCM microcapsules is mostly taken into account by using the heat capacity method [119]. In the heat capacity method, the contribution from the latent heat due to the phase change process is considered by using a piecewise temperature dependent function for the specific heat capacity of the PCM [89,120,121]:

(5) ρcp,c(T)={ρcp,cfor T<TPC−ΔTPC2ρcp,c+hfmpcmΔTPCfor TPC−ΔTPC2 ≤T≤TPC+ΔTPC2ρcp,cfor T>TPC+ΔTPC2

Here, *c_p,c_* is the specific heat capacity of concrete (J/kg K), *h_f_* the heat of fusion of the PCM, *m_pcm_* the quantity of PCM per cubic meter of concrete (kg), *T_PC_* the phase change temperature, Δ*T_PC_* the temperature window. Using this approach, the phase change can be simply simulated. Other approaches, such as the “‘Heat Transfer with Phase Change” approach, are seldom used [122,123].

**Figure 29:**
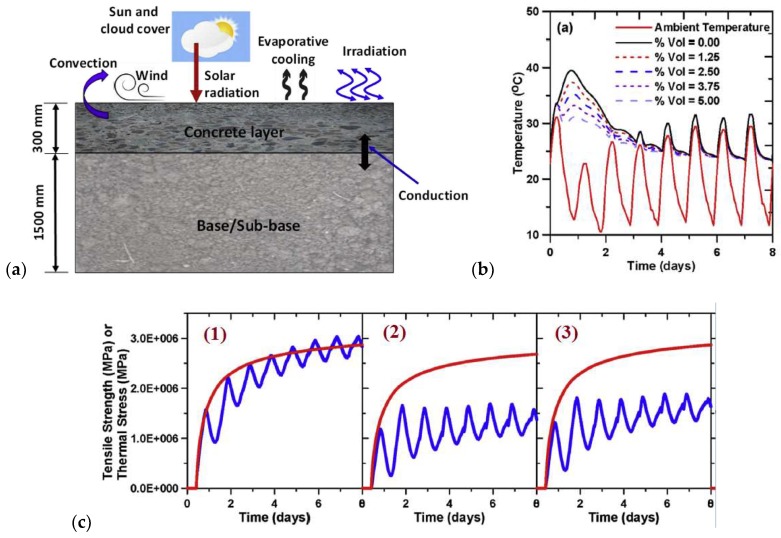
(**a**) Schematic model of a concrete pavement subjected to environmental effects at early ages; (**b**) comparison of maximum section temperatures for the case when PCM replaces a part of the cement paste; (**c**) critical thermal stress and tensile strength development for: (1) OPC concrete, (2) concrete with 10% PCM replacing cement paste, and (3) concrete with 10% PCM replacing fine aggregate. The monotonically increasing curve corresponds to the strength development. The pavement is placed in Phoenix, AZ in in the month of July, and the PCM has a phase transition temperature around 35 °C [124].

Young et al. [90] developed a one-dimensional finite difference model for temperature development in early-age concrete pavements. The model showed that the addition of 10 vol% microencapsulated PCM within the pavement can induce noticeable reductions in the temperature developed within the first 24 h of placement. Furthermore, the addition of PCM also reduced local temperature gradients developed within the pavement section so long as the effective thermal conductivity of the pavement was not reduced. Arora et al. [124] further extended the finite-difference approach to take strength and stress development into account. As already discussed, the addition of PCM may have detrimental effects on the concrete strength, and these effects should be considered simultaneously with the temperature effects. They analyzed a concrete pavement exposed to the environment, as shown in Figure 29a. In Figure 29b, it can be seen that the addition of PCM microcapsules is able to reduce the peak temperature. Their analysis showed that, if properly designed (i.e., if the appropriate amount of PCMs with the appropriate phase change temperature are used), the PCM-concrete mix is able to reduce the stress during the hardening phase and thereby prevent formation of thermal cracking, as shown in Figure 29c.

**Figure 30:**
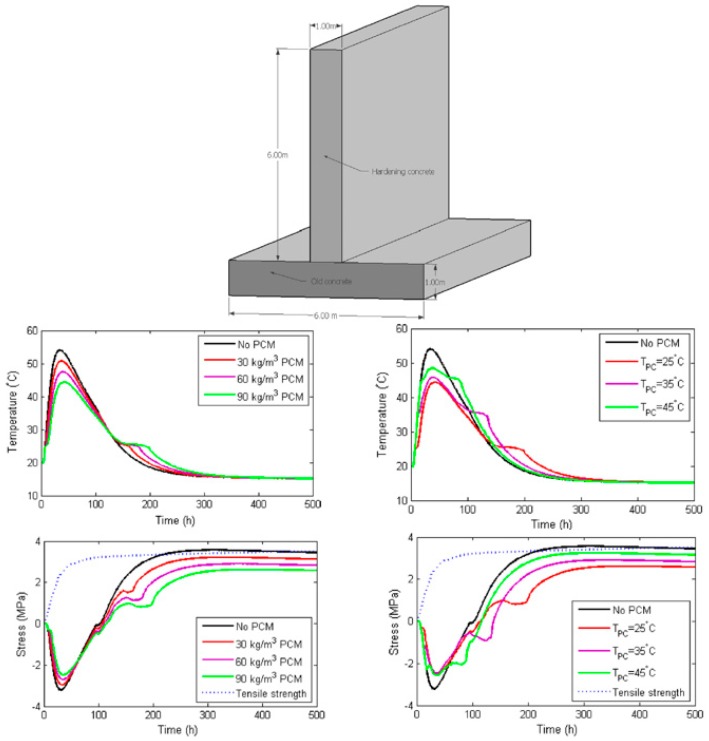
Wall-slab system considered in the analysis, together with temperature and stress developments for various parameters [120].

Šavija and Schlangen [120] used a commercial FE package and the heat capacity method to simulate temperature and stress development in a hardening concrete wall cast on an existing slab (Figure 30). This model considers both the temperature and stress development and can take time dependent material properties into account. This enables the user to tailor their PCMs (in terms of quantity, phase change temperature which can be selected based on environmental conditions and heat of fusion) for the application.

Based on an example structure analyzed in their study, they concluded that:

- In semi-adiabatic (i.e., field) conditions, the addition of PCM in hardening concrete has potential to delay the temperature rise, reduce the maximum tensile stress, and delay its occurrence. The maximum tensile stress is inversely proportional to the amount of PCM added to the mix (see Figure 30).
- In semi-adiabatic conditions, the phase change temperature does influence the maximum temperature developing in the structure (Figure 30).
- An increase in the latent heat of fusion serves the same purpose as an increase in PCM addition: it lowers the maximum temperature and maximum stress and delays their occurrence. Especially the cooling phase is prolonged. Therefore, a trade-off between the heat of fusion and quantity of PCM microcapsules is possible, where a smaller amount of PCMs with a higher heat of fusion can be used with the same (thermal) efficiency. This would be beneficial also in terms of mechanical properties of the concrete.

Regarding performance of concrete with PCMs for reducing freeze/thaw damage, not many literature references are available. References [22,43] showed that PCMs can potentially be used for reducing the number of freezing/thawing cycles. The only numerical model dealing with this issue has recently been proposed by Esmaeeli et al. [123]. They developed a 1-dimensional finite difference model combined with homogenization techniques to study the influence of PCM impregnated LWAs on the number of freeze/thaw cycles. Their model showed that the transition temperatures of PCM during freezing and melting events are important properties that govern the effectiveness of PCM at reducing the impact of freeze-thaw cycles. In other words, they help control how quickly the temperature of the pavement drops below the freezing point of pore solution within concrete pores. The model results also showed that low transition temperatures were more effective. It has to be noted that the model of Esmaeeli et al. [123] focuses only on temperature effects: the effects of mechanical damage/cracking are not taken into account. Since, as already discussed, the addition of PCMs (especially microencapsulated PCMs) may result in a reduction of concrete strength, these effects need to be considered together with the temperature effects in order to fully assess the effectiveness of PCMs for reducing freeze/thaw damage.

## 6. Summary and Future Research Needs

In this paper, a systematic review of possible uses of phase change materials in concrete technology for smart (i.e., active) crack control is presented. Types of phase change materials and technologies for their use in concrete are described. Major fields of application, i.e., control of early age cracking and freeze/thaw damage in concrete structures, are reviewed. In addition, effects of PCMs on typical parameters of concrete, such as strength and workability, are reviewed. Finally, models for PCM based concrete are presented and discussed. It can be concluded that the use of PCMs for crack control in concrete has shown promising results so far.

Before the widespread application of this technology, however, several aspects deserve further study. First, although research studies so far have shown that the technology is promising, there have been no comparisons with methods used at the moment for crack control (such as e.g., air entrainment used for controlling freeze/thaw damage). In order for asset owners to be interested in the technology, it needs to be shown that it is advantageous compared to current practice. Second, no studies focusing on the long-term durability of the material are available. This information is necessary for performing life-cycle analyses. Finally, cost considerations (especially relatively high costs of microencapsulated PCMs) have been left out in the studies. Although it is expected that the materials would become cheaper if they were to be used on the large scale, it is important to keep in mind that concrete in itself is a relatively cheap building material and large increases in price are, for most contractors and owners, not acceptable.

The technology at the moment has reached a stage where field trials are needed to test its efficiency in practice. In the beginning, it is suggested to make simple structures such as concrete pavements or walls on slab, which bring relatively low risk. If crack control in these trail structures were to be successful, major applications of this technology could be for massive retaining walls (for hydration temperature control) and airport pavements (for reduction of freeze thaw damage and ice accumulation).

## Figures and Tables

**Table 1:** Advantages and drawbacks of various phase change material (PCM) types [26,27,28].

Classification	Advantages	Drawbacks
Organic PCMs	1. Availability in a large temperature range 2. High heat of fusion 3. No supercooling 4. Chemically stable and recyclable 5. Good compatibility with conventional construction materials	1. Low thermal conductivity 2. Relatively large volume change 3. Flammable
Inorganic PCMs	1. High heat of fusion 2. High thermal conductivity 3. Low volume change 4. Low cost 5. Sharp phase change 6. Non-flammable	1. Corrosive to metals 2. Supercooling
Eutectics	1. Sharp melting point 2. Properties can be tailored to match specific requirements 3. High volumetric thermal storage density	1. Limited data on thermo-physical properties for many combinations 2. High cost
